# Alterations of Nutritional Status in Children and Adolescents with Acute Lymphoblastic Leukemia

**DOI:** 10.3390/children11030334

**Published:** 2024-03-11

**Authors:** Izabela Kranjčec, Ines Pranjić, Jelena Roganović, Maja Pavlović, Nada Rajačić, Sara Sila

**Affiliations:** 1 Department of Oncology and Hematology, Children’s Hospital Zagreb, 10000 Zagreb, Croatia; jelena.roganovic@kdb.hr (J.R.); mpavlovic@kdb.hr (M.P.); nada.rajacic@kdb.hr (N.R.); 2 Croatian Academic Centre for Applied Nutritional Science, 10000 Zagreb, Croatia; ines.pranjic123@gmail.com; 3 Referral Center for Pediatric Gastroenterology and Nutrition, Children’s Hospital Zagreb, 10000 Zagreb, Croatia; sara.sila@kdb.hr

**Keywords:** leukemia, acute lymphoblastic, children, nutritional status, anthropometry, body mass index

## Abstract

Malnutrition is often observed in pediatric cancer patients and has been recognized as a risk factor for relapse and survival. Maintaining an appropriate nutritional status during anticancer treatment has, therefore, been more and more frequently perceived as an additional requirement for optimal therapy outcomes. The aim of our study was to establish alterations of nutritional status in 26 children and adolescents treated for acute lymphoblastic leukemia (ALL) at the Children’s Hospital in Zagreb, Croatia, between 2016 and 2021, by using anthropometric measures and serum albumin levels. The majority of patients (53.8% female, median 4 years, 52.2% intermediate-risk leukemia group) had normal weight at the beginning of chemotherapy. The percentage of overweight/obese patients increased from 4.2% at diagnosis to 37.5% at the end of intensive therapy. Apart from a significant increase in body weight (BW) and body mass index (BMI) for age, a notable decline in body height/body length (BH/BL) for age in the observed period was recorded, especially in high-risk leukemia patients. The alterations in serum albumin values were not significant, nor was their correlation with BMI. Dietary consultation was offered to all patients, while children with a decline in BMI and BH/BL received additional nutritional support.

## 1. Introduction

Recent advances in the treatment of acute lymphoblastic leukemia (ALL), the most common type of pediatric malignancy, have mostly been attributed to risk stratification and risk-adapted chemotherapy protocols, the development of novel, targeted drugs, and improved supportive therapy [1]. Survival rates exceeding 80% in developed countries have, therefore, been reached by careful infection control, efficient pain management, and better emesis prevention [2,3]. Moreover, maintaining appropriate nutritional status during cancer management is more and more frequently perceived as a supplementary prerequisite for optimal treatment outcomes [4].

Malnutrition in children, defined as both undernutrition and overnutrition, has been recognized as a risk factor for decreased overall survival and higher relapse rates, with a negative impact on susceptibility to infection, drug pharmacokinetics, and quality of life [5,6]. Although undernutrition in children at the time of malignant disease diagnosis is more frequently observed in low- and middle-income countries and in patients with solid tumors, a few studies from developed countries have reported on inadequate nutritional status in children with ALL, with a significant proportion more often being categorized as overweight and obese [7,8,9,10].

Screening for nutritional problems and continuous assessment of nutritional status with adequate tools are the cornerstones of tackling malnutrition in children with cancer [11]. To alleviate intolerance to treatment and improve prognosis in general, dietary counseling should be offered to all patients, with enteral supplements and parenteral support introduced in selected cases [12].

The aim of our study was to determine the nutritional status of children and adolescents with ALL at specific time points of intensive and maintenance chemotherapy, establish the alterations of anthropometric values and serum albumin levels in the course of ALL management, and record the nutritional measures undertaken during treatment.

## 2. Materials and Methods

This observational retrospective study included all patients newly diagnosed with ALL, 26 children and adolescents (14 girls and 12 boys), aged 0–17 years, treated at the Department of Oncology and Hematology, Children’s Hospital Zagreb, Croatia, during a six-year period (1 January 2016–31 December 2021). Epidemiological, clinical, and anthropometric data were extracted from the patients’ electronic medical records.

Epidemiological data included age, sex, and year of diagnosis. Clinical data comprised the duration of the signs and symptoms of the disease, the treatment protocol used, assigned risk stratification group (standard, intermediate, high), duration of intensive chemotherapy (months), and nutritional status. Nutritional status was determined by using the following anthropometric measures: body weight (BW), body height (BH) or body length (BL), and body mass index (BMI). BMI was calculated according to the formula: BW in kilograms divided by the square of the height in meters. BW for age z-score, BH/BL for age z-score, and BMI for age z-score were determined using the World Health Organization (WHO) growth reference data [13] for children up to 5 years of age and Centers for Disease Control and Prevention (CDC) reference data for children older than 5 years of age [14]. Additionally, serum albumin levels were noted.

**Figure 1:**
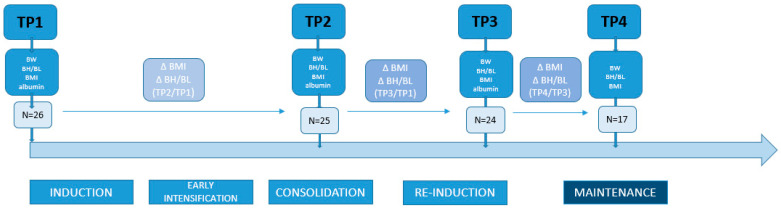
Study design. Abbreviations: TP—time point; BH—body height; BL—body length; BW—body weight; BMI—body mass index.

Nutritional status was determined at four time points: 1. at diagnosis (TP1), 2. in the middle of intensive chemotherapy (during consolidation therapy) (TP2), 3. at the end of intensive chemotherapy (TP3), 4. a year after the end of intensive chemotherapy (during maintenance therapy) (TP4). Changes in BH/BL for age z-score and BMI for age z-score were determined at TP2 (subtraction of variable at TP2 from variable at TP1), at the end of intensive treatment (subtraction of variable at TP3 from variable at TP1), and one year after the end of intensive treatment (subtraction of variable at TP4 from variable at TP3) in all participants. Albumin levels were determined at TP1, TP2, and TP3. The study design is depicted in Figure 1.

Nutritional interventions, including nutritional consultation, placement of nasogastric tube, and enteral (EN) and parenteral (PN) nutrition, were also documented.

The primary objective of this study was to determine the nutritional status of children and adolescents with ALL at specific time points (TP1 to TP4) of first-line chemotherapy and to describe the changes in their nutritional status during intensive treatment and maintenance therapy. Alterations in nutritional status with regard to sex and risk group, as well as the provision of nutritional support, were also recorded.

The normality of data was assessed by the Shapiro–Wilk test. Numerical data were presented as median (min–max) and categorical data as N (%). Differences in non-categorical data were assessed using the Mann–Whitney-U test or the Kruskal–Wallis test. The Friedman test was used for repeated measures of z-scores for BW, BH, BMI, and albumin levels. Spearman’s rank order correlation was performed to test for correlation between albumin levels and BMI for age z-score. A *p*-value < 0.05 was considered statistically significant in two-sided tests.

## 3. Results

The median age in our cohort was 4.0 years (range 0.8–14.9). The majority of patients (38.5%) were diagnosed in the year 2018 after a median of 1.5 months (range 0.8–3) of signs and symptoms of the malignant disease. Apart from the two infants that were initially treated according to the Interfant-06 protocol, all other children were treated according to the ALL-IC BFM 2009 protocol, which includes intensive chemotherapy (induction, early intensification, consolidation, and re-induction) and maintenance therapy, with a median duration of intensive chemotherapy of 8 months (range 1–12).

Induction chemotherapy for all patients included four-week prednisone therapy (60 mg/m^2^) with nine-day tapering along with three cytostatics, while the glucocorticoid used in re-induction for all patients was dexamethasone (10 mg/m^2^) over three weeks, followed by a nine-day withdrawal. Five-day dexamethasone (20 mg/m^2^) was a glucocorticoid base of all six high-risk blocks, the consolidation therapy administered to high-risk patients, while standard- and intermediate-risk patients received no steroid therapy during consolidation.

Most of the patients were stratified as intermediate-risk (52.2%), followed by a standard- (26.1%) and high-risk group (21.7%), and all had negative central nervous system status (CNS1). Four patients died, including both infants in the later course of treatment after the first relapse during second-line therapy due to severe infection (invasive fungal infection and multi-resistant Pseudomonas sepsis). Two other patients died due to severe infection resulting in multi-organ failure during first-line therapy (one in induction, the other in re-induction). In two patients, allogeneic stem cell transplantation was performed (one in upfront and one in second-line therapy). Considerable variability in treatment duration (range 1–12 months) was, therefore, due to early deaths, refractoriness to therapy, and adverse events associated with anticancer medications. Twenty-two patients (84.6%) are alive and in remission, with a median follow-up of 5.3 years (range 3.0–8.1). Patient characteristics and the course of chemotherapy are presented in Supplementary Table S1.

Table 1 shows the nutritional status of participants at different time points. A statistically significant increase in BMI for age z-score from the start to the end of intensive treatment, with a decrease following one year into maintenance therapy, was observed (*p* = 0.001). BH for age z-score decreased significantly during the treatment period and continued to decrease one year after the end of intensive chemotherapy (*p* ≤ 0.001). Albumin levels did not differ significantly between different time points (*p* = 0.695). There was a weak, non-significant correlation between albumin levels and BMI for age z-score at TP1 (r = 0.049, *p* = 0.833), TP2 (r = −0.151, *p* = 0.658), and TP3 (r = −0.129, *p* = 0.621).

**Figure 2:**
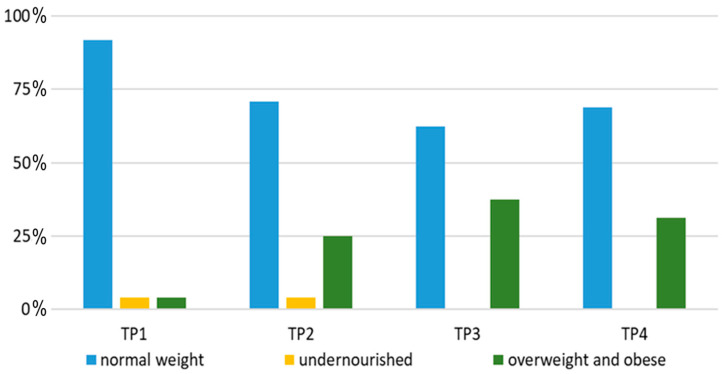
Graphical representation of nutritional status of patients at time points of interest. Abbreviations: TP—time point.

According to the WHO criteria [13], 4.2% of patients were undernourished at the beginning of the treatment, an equal percentage of patients were overweight, while all the others had normal BW (Figure 2). By the end of intensive treatment, the percentage of overweight and obese patients increased to 37.5%. A decrease in the number of obese patients was visible one year into maintenance therapy.

**Figure 3:**
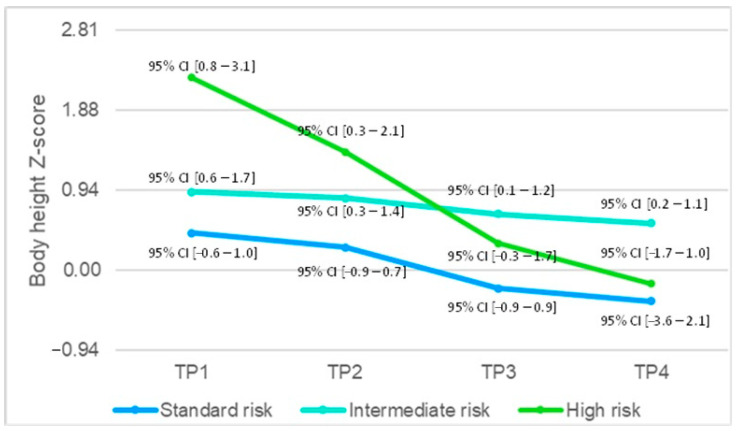
Body height/length z-score at different time points between standard-, intermediate- and high-risk groups. Abbreviations: TP—time-point.

Patient variables were compared with regard to risk group stratification. The high-risk group had significantly higher (1.22 (0.81–1.52)) BW for age z-score at TP1 as compared to the intermediate- (0.4 [−1.41–1.37]) and standard-risk groups (−0.065 [−1.29–1.05]) (*p* = 0.017). There was no statistically significant difference in BW for age z-score at other time points. BH/BL for age z-score differed significantly at TP1 (*p* = 0.025), at TP2 (*p* = 0.042), and at TP4 (*p* = 0.025) between the three groups (Figure 3), with the high-risk group having a significantly higher BH/BL for age z-score (2.26 [0.95–3.05] at TP1, 1.39 [0.47–1.94] at TP2, and −0.16 [−0.98–0.04] at TP4) as compared to children in the intermediate- (0.92 [0.07–3.21] at TP1, 0.85 [−0.13–2.62] at TP2, and 0.55 [−0.19–1.52] at TP4) and standard-risk (0.44 [−1.13–1.05] at TP1, 0.27 [−1.46–0.65] at TP2, and −0.36 [−2.03–0.19] at TP4). There was a statistically significant difference in BMI for age z-score at TP4 (1.04 [0.8–2.05] for standard, 0.12 [−1.23–1.49] for intermediate, and 1.05 [0.92–1.63] for high risk) between the three groups (*p* = 0.025). Other parameters (age and BW, BH/BL, and BMI at other time points) did not differ significantly between risk groups.

In addition to risk group, differences in individual parameters by gender were also observed. BH for age z-scores of female and male patients differed significantly at TP1 (*p* = 0.02) and at TP2 (*p* = 0.03), with significantly higher BH for age z-score in girls (1.62 [0.07–3.05] at TP1, 0.94 [−0.13–1.94] at TP2) as compared to boys (0.59 [−1.13–3.21] at TP1, 0.28 [−1.69–2.62] at TP2). The median BMI for age z-score increased from the beginning to the end of treatment in both girls and boys, with a decrease in BMI for age z-score one year after the end of treatment. No difference in change of BH/BL z-score or BMI z-score was observed between boys and girls.

Fifteen (57.7%) patients received supplementary support during the course of the treatment, in the form of EN or PN. Of those who received nutritional support, the majority (73.3%) experienced weight loss, while the rest (26.7%) experienced significant weight gain and/or hypoglycemia. Eight (30.8%) patients received EN via nasogastric tube and/or parenteral (PN) feeding at some point during the treatment.

BMI for age z-score of children who received EN or PN was significantly lower at TP2 (−0.05 [−0.63 to 1.1]) as compared to those who did not (+0.84 [−1.9 to 2.06]) (*p* = 0.04). Moreover, patients who received nutritional support experienced a median −1.02 (−2.73 to −0.58) decrease in BH/BL for age z-score at TP3, as opposed to patients who did not require nutritional support (−0.4 [−1.47 to 0.34]) (*p* = 0.015). There was no difference in BW for age z-score, BH/BL for age z-score, or BMI for age z-score nor was there a change in BMI for age z-score and BH for age z-score at specific time-points.

## 4. Discussion

The aim of our study was to demonstrate changes in the nutritional status of pediatric patients treated for ALL from diagnosis up to one year into maintenance therapy. Our study showed that BMI for-age z-score increased significantly from the start to the end of intensive treatment in children with ALL and remained high one year into maintenance therapy. By the end of intensive treatment, the percentage of overweight and obese patients increased. Simultaneously, BH for age z-score decreased significantly during the treatment period and continued to decrease one year after the end of intensive chemotherapy, with the high-risk group experiencing the most significant decline in BH/BL for age z-score.

Malnutrition is observed in up to half of the children and adolescents with cancer worldwide, both during active treatment and follow-up [15,16,17]. Over- and undernutrition in pediatric oncology patients has been linked to the type of malignancy, treatment intensity, and socio-economic status, including parents’ educational level [16,18].

At the time of diagnosis, children with solid tumors are more likely to be undernourished, with up to one-third of the patients affected [19,20,21,22]. However, a severely depleted nutritional status has likewise been reported among newly diagnosed ALL patients, mainly in underdeveloped countries; for example, the proportion of undernourished ALL patients in India is 88% [23,24,25]. In high-income countries, the majority of ALL patients have normal BMI at diagnosis [26], which we also observed in our study. However, patients with ALL in high-income countries are more likely to experience overnutrition in the course of the treatment. A prospective study on 133 Dutch children diagnosed with hematological and solid malignancy established an increase in BMI in the first three months of treatment, a rising trend continuing into the following months and resulting in a redoubling of the proportion of overweight/obese patients [27]. A rapid increase in BW, BMI, and fat mass (FM) was detected among South African children with hematological neoplasms during the first few months of chemotherapy [28]. Similar concerning nutritional alterations, even more pronounced, were observed in our cohort, with the proportion of overnourished patients increasing by more than one-third three months into the treatment. A statistically significant risk of overweight and obesity in adolescents and young adult (AYA) ALL patients, compared to other tumor types, also extends into survivorship [29]. This was also confirmed in a meta-analysis [29] showing a mean BMI z-score of 0.83 SD (corresponding to the 60th BMI percentile) in pediatric ALL survivors. Although our data indicate a slight improvement in obesity rates one year after the end of intensive therapy, they remained high and did not restore to baseline values.

In our cohort, both girls and boys experienced an increase in BMI z-score, along with a significant decrease in BH/BL z-score. However, due to the small number of patients included in this study, we could not test for differences according to age. Nevertheless, the high prevalence of obesity in ALL survivors was shown to be independent of patient- and treatment-related characteristics, emphasizing the need for early nutritional intervention in all ALL patients [30].

Corticosteroids are known to play critical roles in regulation of energy intake, storage, and mobilization. Previous studies reported significantly higher energy intakes in pediatric ALL patients treated with corticosteroids [31,32]. It is still not clear whether there is a dose effect of corticosteroids on obesity in ALL patients [29]. The potential impact of cancer treatment on energy balance may last beyond the completion of treatment and may even become permanent. A few studies suggest that young age at diagnosis is associated with a high prevalence of obesity in ALL survivor; however, the evidence remains inconclusive [29].

Unlike in adults, chronic diseases in children can impact their growth and development and consequently limit their potential to achieve their target height. Short stature is a well-known complication of ALL treatment [30], especially evident in patients who have undergone cranial radiotherapy (CRT) [29,33]. In our cohort, growth was significantly impaired at the end of intensive chemotherapy and, more importantly, continued to decline one year into maintenance therapy. Our results are in accordance with previous studies [30,34], which demonstrated a decrease in height *Z*-score during therapy, with partial improvement during the off-therapy period. However, Browne et al. showed that while height z-score improved during the 5-year off-therapy period, it never returned to the levels at diagnosis [34]. In the same study, standard- and high-risk status were associated with lower height z-score during intensive chemotherapy. Although our patients in the high-risk group had significantly higher height z-scores at diagnosis, a remarkably more prominent decline in height z-scores was evident in this group.

Nutritional status, alongside socio-economic background, has been acknowledged as a prognostic factor of treatment outcome in children with ALL more than two decades ago [35], and these findings were confirmed by multiple studies over the years for all types of pediatric cancer [21,36,37,38]. A critical analysis of more than a thousand children with ALL supports the evidence of undernutrition as an adverse prognostic factor in the long-term outcomes of childhood hematological malignancies [39]. Nosocomial infections were more frequent among poorly nourished ALL patients [24,40]. Abnormal nutritional status seems to play a more significant role in survival in solid tumors. A systematic review by Joffe and colleagues verified poorer outcomes in children and adolescents with Ewing sarcoma, osteosarcoma, and rhabdomyosarcoma [36]. Overnourished children with leukemia suffer from up to a 50% higher risk of relapse and death [37].

To date, there is no consensus on optimal nutritional assessment tools in children with cancer, yet it is well established that complete evaluation requires clinical information, biochemical indices, anthropometric values, and dietary intake history [41]. 

In children and adolescents in general, anthropometry, such as weight-for-height index, is recommended by WHO as a reliable nutritional assessment method [42]. The International Society for Paediatric Oncology (SIOP), Committee on Paediatric Oncology in Developing Countries (PODC), and their Nutrition Working Group (NWG), suggest using a standard nutritional evaluation protocol in pediatric oncology that is easily administered and cheap, and therefore can also be incorporated in low- and middle-income countries’ health care systems [43]. Apart from the application of BW, BH/BL, and BMI measurements and WHO growth chart percentiles or Z-scores for age, mid-upper arm circumference (MUAC) and triceps skinfold thickness (TSFT) measurements are recommended [43].

Many authors emphasize the value of arm anthropometry such as MUAC and TSFT, which are considered sensitive indices independent of tumor mass, in order to avoid the underestimation of malnutrition prevalence. The first recommendations date from the late 1990s [44,45,46,47,48]. Although arm anthropometry, according to abundant published data, assists in undernutrition recognition during pediatric cancer treatment, some studies have failed to present sufficient evidence in favor of its use in survivorship [49]. However, it is still evidently insufficiently utilized not only among our patients but also in other developed European countries, such as the United Kingdom [50].

Dual-energy X-ray absorptiometry (DEXA) is perceived as a gold-standard body composition measure in a hospital setting [51,52]. However precise, it cannot be utilized in all children in all phases of treatment due to the occasional deterioration of patients’ general condition and safety issues regarding radiation dosage [53,54]. Moreover, well-trained personnel are required for this rather costly examination, all valid reasons why it could not have been employed at our site.

Serum albumin/prealbumin are considered adequate laboratory markers of nutritional status in cancer patients, with a correlation to outcome [55,56]. Serum prealbumin can be utilized to extricate patients at risk for malnutrition, as shown by Yaprak and colleagues [48]. However, a recent study by Garofolo and associates found no association between serum albumin and anthropometric measures [57], as was demonstrated in our study.

Given our small-scale sample size and excellent survival rates, the influence of nutritional status on outcome was not studied. The survival rate in our cohort was only 2% below The Organization for Economic Cooperation and Development (OECD) countries average [58]. Four patients died during ALL treatment. Three of them were well-nourished and remained so during treatment. Only one patient was overweight at diagnosis and had an increase in BMI during the treatment period but did not become obese.

The patients with the most prominent deterioration in nutritional status had received nutritional intervention in the form of nutritional counselling, EN, or PN. Children who gained significant amounts of weight were advised to limit their intake of high-energy, high-sugar foods. It is unclear whether lifestyle interventions are successful in the prevention of obesity in these patients. Recent meta-analyses showed that lifestyle interventions did not lead to significant changes in BMI-related measures [59]. However, in a pilot study by Walters et al. [60], twenty-three participants with newly diagnosed ALL were instructed to follow a low-glycemic diet. The majority of patients were able to adhere to the recommended diet, and, importantly, the authors did not observe an increase in BMI z-score during the induction period or over the 6-month intervention period. Nevertheless, larger clinical trials with longer follow-up periods are required to make definitive conclusions and recommendations for lifestyle interventions in ALL patients.

A few studies have demonstrated that weight changes during intensive chemotherapy might have a more significant impact on the outcome than the initial BW status [61]. Our study provides no data on survival in relation to anthropometric indices but accentuates the problem of obesity arising during pediatric ALL treatment, which might be the greatest strength of our investigation. Our study’s limitations are the retrospective nature of the study, single-institution design, and small-scale study sample. Furthermore, due to the retrospective design, we could not estimate the energy and nutritional intakes of patients, and anthropometric measures were the only available parameters for nutritional status assessment. Whether BMI charts are the most appropriate nutritional evaluation method in pediatric oncology is still a matter of debate. The distinction between fat tissue and muscle in children and adolescents with cancer cannot be determined using BMI [49]. However, in most published surveys, BMI served as a measure to define obesity. Thus, since emerging overnutrition in children during ALL treatment was the main finding of our study, using nutritional status parameters might seem appropriate [38]. We plan to perform a prospective survey on alterations of nutritional status in children and adolescents treated for ALL in all pediatric oncology centers in Croatia by applying the entire recommended nutritional assessment set (anthropometric measures, laboratory parameters, dietary intake questionnaires) to obtain detailed insights into nutritional status changes and provide tailored nutritional interventions to improve outcomes.

## 5. Conclusions

Children and adolescents treated for ALL are at increased risk for obesity and simultaneously experience retarded growth. The decline in growth observed during intensive treatment also continues into the maintenance phase. Nutritional interventions should be undertaken in all pediatric patients in order to maintain appropriate nutritional status, avoid negative consequences of overnutrition, minimize growth impairment, and optimize treatment outcomes. Further prospective, preferably multi-centric research on this topic on a larger cohort of pediatric ALL cases is needed and should utilize a complete set of nutritional status assessment tools.

## Figures and Tables

**Table 1:** Alteration of anthropometric measurements in subjects at observed time points.

	TP1 (*n* = 26)	TP2 (*n* = 25)	TP3 (*n* = 24)	TP4 (*n* = 17)	*p*-Value
BW for age z-score (SD), median (min–max)	0.58 (−1.41–1.52)	0.79 (−2.22–2.66)	0.83 (−1.43–2.51)	0.37 (−0.61–1.44)	0.027
BH/BL for age z-score (SD), median (min–max)	0.94 (−1.13–3.21)	0.59 (−1.69–2.62)	0.42 (−2.18–2.11)	0.19 (−2.03–1.85)	<0.001
BMI for age z-score (SD), median (min–max)	0.05 (−2.75–1.32)	0.49 (−3.82–2.92)	0.76 (−1.64–2.68)	0.55 (−1.23–2.05)	0.001
Albumin (g/L), median (min–max)	37 (29–46)	36 (23–43)	36 (22–41)	N/A	0.695

Abbreviations: TP—time-point; BH—body height; BL—body length; BMI—body mass index; BW—body weight. Albumin (g/L) was unavailable for 3 out of 26 (11.5%) of patients at TP1, 13 out of 25 (52.0%) of patients at TP2 and 7 out of 24 (29.2%) of patients at TP3.

## Data Availability

The complete data presented in this study are available on request from the corresponding author. The data are not publicly available due to ethical issues.

## Supplementary Materials

The following supporting information can be downloaded at: https://www.mdpi.com/article/10.3390/children11030334/s1, Table S1. Patients’ characteristics and treatment course.
