# Supplementary material for: Alterations of Nutritional Status in Children and Adolescents with Acute Lymphoblastic Leukemia
Source: Children (Basel). 2024 Mar 11;11(3):334. doi: 10.3390/children11030334 (PMC10969479; doi:10.3390/children11030334)
Supplement: Supplementary file 1 [file children-11-00334-s001.zip › children-2896635-supplementary.pdf]

Table S1: Patients' characteristics and treatment course

Patient	Age	Sex	Protocol	Risk	BW1	BH1	BW2	BH2	BW3	BH3	BW4	BH4	Duration	Adverse events	Nutritional intervention	Additional comments
1	7.6	F	ALL IC-BFM	IR	25.5	136	30.4	136	32.5	138	30.5	146	7	I: mild mucositis; RI: febrile neutropenia, seizures	DC	
2	3.8	M	ALL IC-BFM	SR	16.2	104	19	104	20	104	21	112	6	I: mild mucositis; C: transaminitis; RE: mild mucositis, transaminits, febrile neutropenia	DC	
3	0.8	M	Interfant	N/A	9.5	72	9.4	72	10.7	75	12.4	82	8	I: febrile neutropenia, urinary infection; C: sepsis, IFI; RE: febrile neutropenia	DC, EN	Congenital anal atresia; relapse; death due to IFI during the 2nd line therapy
4	4.2	F	ALL IC-BFM	IR	17	110	18	110	18	110	20.8	116	7	I: mild mucositis; EI: febrile neutropenia, pneumonia; C: mild transaminitis; RI: febrile neutropenia, pneumonia, IFI	DC	ICU due to pneumonia
5	5.2	F	ALL IC-BFM	HR	23.4	120	20.6	120	27.5	122	30	124	12	I: febrile neutropenia, sepsis, IFI, mucositis; EI: febrile neutropenija, sepsis, IFI, Clostridium enterocolitis; C: febrile neutropenia, seizures, RI: febrile neutropenia	DC, EN, PN	ICU due to sepsis and seizures
6	2.5	F	ALL IC-BFM	IR	13	94	14.4	97	15.6	97	16.8	106	7	I: febrile neutropenia, mild mucositis, hypoglycemia; RI: febrile neutropenia, sepsis, ileus, pancreatitis	DC, EN, NGS	ICU due to sepsis

7	3.5	M	ALL IC-BFM	IR	13.1	102	14.6	103	17	103	*	*	7	IE: febrile neutropenia; C: febrile neutropenia, urinary infection; RI: febrile neutropenia, urinary infection	DC	
8	1.3	F	ALL IC-BFM	IR	10.5	78	10.7	78	10.7	82	N/A	N/A	6	I: acute tumor lysis syndrome, febrile neutropenia, sepsis, mild mucositis; EI: febrile neutropenia; C: febrile neutropenia, Clostridium entercolitis, IFI, mild mucositis, transaminitis; RI: febrile neutropenia, sepsis, pneumonia	DC, EN, PN, NGS	ICU due to sepsis; death due to multiorgan failure at the end of re-induction
9	2.2	M	ALL-IC BFM	N/A	13	91	N/A	N/A	N7A	N/A	N/A	N/A	1	I: febrile neutropenia, sepsis, pancreatitis	DC, EN, NGS	ICU due to sepsis and pancreatitis; death due to multiorgan failure at the end of induction
10	5.2	F	ALL IC-BFM	SR	20.7	119	21.8	119	23.8	124	*	*	6	I: liver injury; EI: febrile neutropenia, sepsis, mild pancreatitis; RI: febrile neutropenia, urinary infection, transaminitis	DC	ICU due to sepsis
11	8.6	M	ALL IC-BFM	IR	33.3	140	41.8	142	43.5	143	*	*	8	I: febrile neutropenia, mild mucositis, transaminitis; IE: transaminitis, pelvic joint pain; C: mild mucositis, transaminitis; RI: febrile	DC	

														neutropenia, sepsis, Clostridium enterocolitis, mild mucositis, transaminitis		
12	3.9	M	ALL IC-BFM	SR	16.8	107	18.2	107	22	107	*	*	6	I: febrile neutropenia; RI: febrile neutropenia	DC, PN	
13	7.3	M	ALL IC-BFM	HR	28.4	131	33.5	131	34.2	131	36.5	138	9	IE: febrile neutropenia; C: febrile neutropenia, severe mucositis, acute abdomen, transaminitis; RI: febrile neutropenia, PRES	DC	ICU due to PRES
14	7.2	F	ALL IC-BFM	HR	30	130	30	130	27.8	130	32	130	10	I: febrile neutropenia, sepsis, cerebral ischemia, mucositis; C: febrile neutropenia, sepsis, IFI, mucositis, transaminitis; RI: febrile neutropenia, sepsis, IFI; mucositis, transaminitis	DC, EN, PN, NGT	ICU due to cerebral ischemia, sepsis and IFI
15	4	F	ALL IC-BFM	IR	17.6	107	19.5	112	21	112	22.5	119	7	IE: febrile neutropenia, pneumonia, IFI, tranaminitis; RI: febrile neutropenia, mild mucositis, transaminitis	DC, EN	
16	5.5	F	ALL IC-BFM	HR	26	126	26	126	N/A	N/A	N/A	N/A	5	I: febrile neutropenia, sepsis, pneumonia, IFI, PRES; C: febrile neutropenia, transaminitis	DC	ICU due to PRES; allogeneic hematopoietic stem cell transplant during 1st line chemotherapy
17	0.8	F	Interfant	N/A	10.3	78	10.8	80	10.8	81	15.3	96	6	I: febrile neutropenia, sepsis, mild mucositis; C: febrile neutropenia, sepsis, severe mucositis	DC, EN, PN	Relapse; death due to sepsis during

																the 2nd line therapy
18	7.6	F	ALL IC-BFM	IR	22.9	128	26.4	129	29.2	129	31.5	137	7	I: febrile neutropenia, sepsis, mild mucositis, transamintis; EI: febrile neutropenia, sepsis; RI: febrile neutropenia, mild mucositis, seizures	DC	ICU due to seizures
19	8	F	ALL IC-BFM	HR	36	144	33.5	144	33.3	144	*	*	11	I: febrile neutropenia, urinary infection, Clostridium enterocolitis, mild mucositis; EI: febrile neutropenia, sepsis, seizures; C: febrile neutropenia, sepsis, mild mucositis; RI: febrile neutropenia, sepsis, pneumonia, mucositis	DC, EN, PN	ICU due to seizures and sepsis
20	3.1	M	ALL IC-BFM	SR	14.4	93	15	94	15.9	98	17.1	100	7	I: febrile neutropenia, sepsis, mild mucositis, transaminitis, thrombosis; EI: febrile neutropenia, sepsis; C: febrile neutropenia, Clostridium enterocolitis; RI: febrile neutropenia, sepsis, Clostridium enterocolitis, IFI	DC, EN	ICU due to sepsis and IFI
21	2.9	M	ALL-IC BFM	SR	16.4	99	21.4	99	22	103	20.5	109	6	EI: febrile neutropenija, sepsis, IFI; RI: febrilne neutropenia, mild mucositis	DC	ICU due to IFI
22	3.1	M	ALL-IC BFM	IR	17	101	17.4	102	17.4	104	19	110	6	I: febrile neutropenia, mild mucositis, mild pancreatitis; EI: febrile neutropenia; C:	DC, EN	

														febrile neutropenia, sepsis; RI: febrile neutropenia		
23	4.1	F	ALL IC-BFM	IR	20	112	23.8	112	26.4	113	26	119	7	I: febrile neutropenia, mild mucositis, transaminitis; RI: febrile neutropenia, mild mucositis	DC	
24	3.4	M	ALL IC-BFM	IR	18.5	113	21.5	113	21.8	113	22.5	118	6	I: febrilne neutropenia, mild mucositis; EI: sepsis; RI: febrilne neutropenia, sepsis, Clostridium enterocolitis, severe electrolyte disorder	DC	
25	14.8	M	ALL IC-BFM	HR	47	172	42.4	173	50.2	176	*	*	10	I: febrile neutropenia, mucositis, hyperglycemia; C: febrile neutropenia, mucositis, hyperglycemia, acute kidney injury, RI: febrile neutropenia, hyperglycemia, steroid psychosis, iron overload	DC, EN	
26	3.9	F	ALL IC-BFM	IR	18.5	110	21.2	113	21.8	114	20.6	122	7	I: febrile neutropenia; EI: febrile neutropenia, mild mucositis, hypoglycemia; RI: febrile neutropenia, sepsis	DC	

Legend: age (years); sex (F-female, M-male); risk (SR-standard, IR-intermediate, HR-high); BW (kg) and BH (cm), body weight and body height (1-time point 1, 2- time point 2, 3- time point 3, 4- time point 4); duration (of treatment, months); adverse evets (of interest; I- induction, EI- early intensification, C-consolidation, RE- reinduction); nutritional intervention (DC-dietary counselling, EN- enteral nutrition, PN- parenteral nutrition, NGT-nasogastric tube); additional comments (e.g. relapse, death, intensive care (ICU) admission, IFI (invasive fungal infection)…), N/A- not applicable; *- data missing
